# Decorin-inducible Peg3 Evokes Beclin 1-mediated Autophagy and Thrombospondin 1-mediated Angiostasis*

**DOI:** 10.1074/jbc.M116.753632

**Published:** 2017-02-07

**Authors:** Annabel Torres, Maria A. Gubbiotti, Renato V. Iozzo

**Affiliations:** From the Department of Pathology, Anatomy, and Cell Biology and the Cancer Cell Biology and Signaling Program, Kimmel Cancer Center, Sidney Kimmel Medical College, Thomas Jefferson University, Philadelphia, Pennsylvania 19107

**Keywords:** angiogenesis, autophagy, Beclin-1 (BECN1), cell biology, cell motility, decorin, proteoglycan

## Abstract

We previously discovered that systemic delivery of decorin for treatment of breast carcinoma xenografts induces paternally expressed gene 3 (Peg3), an imprinted gene encoding a zinc finger transcription factor postulated to function as a tumor suppressor. Here we found that *de novo* expression of Peg3 increased Beclin 1 promoter activity and protein expression. This process required the full-length Peg3 as truncated mutants lacking either the N-terminal SCAN domain or the zinc fingers failed to translocate to the nucleus and promote Beclin 1 transcription. Importantly, overexpression of Peg3 in endothelial cells stimulated autophagy and concurrently inhibited endothelial cell migration and evasion from a 3D matrix. Mechanistically, we found that Peg3 induced the secretion of the powerful angiostatic glycoprotein Thrombospondin 1 independently of Beclin 1 transcriptional induction. Thus, we provide a new mechanism whereby Peg3 can simultaneously evoke autophagy in endothelial cells and attenuate angiogenesis.

## Introduction

Paternally expressed gene 3 (Peg3)^3^ was recently identified in our laboratory as a gene induced in the stroma of breast carcinoma xenografts following systemic delivery of decorin (1), a small leucine-rich proteoglycan with antioncogenic and antiangiogenic properties (2–4). We subsequently discovered that Peg3 is essential for decorin-induced autophagy in endothelial cells (5, 6) and that decorin expression is induced both *in vitro* and *in vivo* by proautophagic stimuli like starvation and mammalian target of rapamycin (mTOR) inhibition (7, 8). Furthermore, Peg3 is also necessary for the induction of endothelial cell autophagy evoked by another matrix constituent, endorepellin (9, 10), the C-terminal fragment of perlecan previously implicated in angiostasis (11–15). Together, these studies show that Peg3 is an important link between soluble matrix molecules and their regulation of a vital cellular process, autophagy (16). However, the precise mechanism of Peg3-evoked autophagy in endothelial cells remains unknown.

Structurally, Peg3, one of only ∼79 imprinted genes in the human genome (17, 18), harbors an N-terminal SCAN domain, which functions as a protein-protein interaction motif allowing Peg3 to homo- or heterodimerize, and an extended C terminus containing 12 C_2_H_2_ Krüppel-like zinc finger domains capable of binding DNA (19–21). Functionally, Peg3 has been implicated in several cellular processes involved in cell growth and development. During gastrulation, Peg3 is first detected in the ectoderm and mesoderm with strong expression in extraembryonic tissues (22). In adult tissues, Peg3 is ubiquitously expressed with the highest levels in brain, skeletal muscle, testis, and ovary (22). In skeletal muscle, the interaction of Peg3 with tumor necrosis factor (TNF) receptor-associated factor 2 induces NFκB nuclear translocation (23) and inhibits myogenesis, leading to cachexia (24). This interaction occurs in a subpopulation of interstitial stem cells where Peg3 modulates caspase activity in response to TNFα and contributes to the loss of muscle regeneration (25). Peg3 expression is also considered a stem cell marker in the epidermis, small intestine, and central nervous system (26). Peg3 promotes apoptosis downstream of p53/c-Myc by associating with Siah1a (Seven in absentia homolog 1a) and stimulating Bax translocation from the cytosol to the mitochondrial outer membrane for the release of cytochrome *c* (27, 28). The apoptotic function of Peg3 is activated in neuronal cells during hypoxia (29). In this cell type, Peg3 is primarily expressed in the nucleus and upon induction affects gene transcription, which in turn stimulates Bax translocation (30).

In agreement with the high expression of Peg3 in the brain and its role in development, *Peg3*^−/−^ mice display abnormal behavior and metabolic disorders (31, 32). Female *Peg3*^−/−^ mice exhibit atypical nurturing behavior, and the pups have stunted growth and impaired suckling, leading to decreased survival (32). Conversely, despite the reduction in nutrient intake, these mice have increased body fat, which may be due to the ability of Peg3 to modulate genes involved in lipid metabolism and adipocyte differentiation (33). However, a recent report has provided evidence against a role of Peg3 in maternal care but favors a more general function for Peg3 in regulating body growth (34).

Unlike in normal Mendelian inheritance, imprinted genes are only expressed by either the maternal or paternal allele as the other is silenced via histone alterations and/or promoter methylation (35). Because only one allele is expressed, imprinting is important in the context of cancer as imprinted tumor suppressor genes are more vulnerable to loss of heterozygosity than genes expressed on both alleles. Unsurprisingly, loss of Peg3 due to hypermethylation of the promoter or loss of heterozygosity has been implicated in several malignancies (36–40). In fact, re-expression of Peg3 in ovarian and glioma cell lines suppresses tumorigenicity *in vitro* and *in vivo* (37, 41, 42). In glioma cell lines, reintroducing Peg3 abrogates Wnt signaling by promoting degradation of β-catenin via the proteasome in a non-canonical pathway that is independent of glycogen synthase kinase 3β (42). Intriguingly, this function of Peg3 appears functionally akin with that of decorin (43). These studies provide evidence that this imprinted gene may function as a *bona fide* tumor suppressor.

As mentioned above, we discovered a novel function for Peg3 as a key regulator of decorin-induced autophagy (5, 6). Decorin is primarily synthesized by fibroblasts, smooth muscle cells, and macrophages (44–47) and is involved in modulating several biological processes including collagen fibrillogenesis, bone and skin homeostasis, vertebrate convergent extension, myogenesis, cancer, and angiogenesis (48–64). Although decorin was initially thought to function as a collagen-binding proteoglycan and thus as a primary regulator of collagen fibrillogenesis (50, 65–69), recent evidence shows that decorin plays a much broader role in the modulation of cell signaling pathways via interactions with growth factors and several receptor tyrosine kinases (70). Decorin functions as a tumor repressor, inhibiting cancer growth, migration, and angiogenesis via down-regulation of the oncogenes Myc, β-catenin (in a glycogen synthase kinase 3β-independent manner), and hypoxia-inducible factor 1, α subunit (43, 47, 71–74).

During the early stages of autophagic induction, decorin non-canonically activates the energy sensor kinase AMPK by promoting phosphorylation of the AMPKα subunit at Thr^172^ (6). Concurrently, decorin attenuates phosphorylation of critical antiautophagic effectors such as the serine/threonine-specific protein kinase Akt, mTOR, and p70S6K (6) downstream of vascular endothelial growth factor receptor 2 (VEGFR2) signaling. Similar to AMPK, Peg3 is essential for endothelial cell autophagy evoked by decorin and represents a novel regulator of autophagy (5). Silencing Peg3 with siRNA abrogates the ability of decorin to induce the autophagic gene *MAP1LC3A* and prevents induction of the Beclin 1 (*BECN1*) gene beyond basal levels (5). Interestingly, knockdown of Peg3 also reduces basal expression of *BECN1*, indicating that the two are closely linked (5).

In the present study, we investigated the role of Peg3 in autophagy and angiogenesis (75). We discovered that *de novo Peg3* expression enhanced Beclin 1 transcription and promoted endothelial cell autophagy. Constitutive expression of Peg3 also inhibited endothelial cell migration and evasion from a 3D matrix and evoked secretion of Thrombospondin 1, suggesting that endogenous levels of Peg3 could concurrently regulate both autophagy and angiogenesis.

## Results

### 

#### 

##### Peg3 Localizes to the Nucleus of PAER2 Cells

**FIGURE 1.**
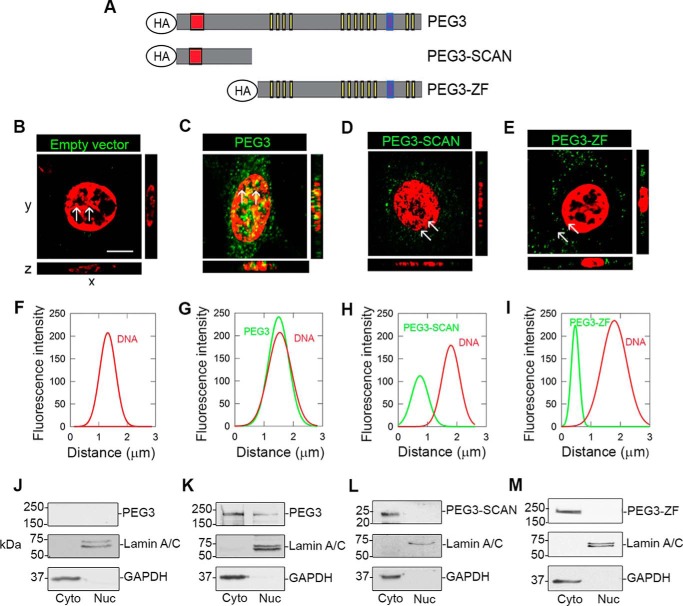
**Peg3 translocates to the nucleus and co-localizes with DNA.** *A*, constructs used for the overexpression of Peg3 and truncations containing either the N terminus and SCAN domain (PEG3-SCAN; *red box*) or C terminus and zinc finger domains (PEG3-ZF; *yellow boxes*). Peg3 also contains a proline-rich region in *blue. B–E*, confocal images of PAER2 cells following a 48-h transfection of Peg3 where an anti-HA antibody was used to detect Peg3 and its truncations (*green*). Nuclei were stained with DAPI to visualize DNA, and the color of the blue channel was changed to *red* for a more efficient visualization of the co-localization (*scale bar*, ∼10 μm). *F–I*, line scan profiles corresponding to each confocal image displaying fluorescence distribution (pixels) measured between the *white arrows* for each channel. All images were captured using the same exposure, gain, and intensity. *J–M*, cytoplasmic (*Cyto*) and nuclear (*Nuc*) fractionations performed after 48-h transfections, validating Peg3 cellular localization.

Peg3 is a putative DNA-binding protein due to its C_2_H_2_ zinc finger motifs (20, 22, 24, 30). Thus, to ascertain whether Peg3 behaves as a DNA-binding protein in endothelial cells, we elucidated its subcellular localization. We transiently transfected PAER2 cells, transgenic porcine aortic endothelial cells overexpressing VEGFR2, with hemagglutinin (HA)-tagged full-length Peg3 or deletion constructs containing either the N-terminal SCAN domain (PEG3-SCAN) or the zinc finger domains (PEG3-ZF) (Fig. 1*A*). The HA tag allowed us to specifically recognize transgenic Peg3 and its truncations. Using confocal microscopy, we found that only full-length Peg3 localized to the nucleus in contrast to both deletions, which remained within the cytoplasm (Fig. 1, *C–E*). Transfection with the empty vector showed no signal (Fig. 1*B*). To validate the presence of Peg3 in the nucleus, we utilized *z*-stack optical sections with *xz* orthogonal views where the yellow color confirms the presence of Peg3 in the nucleus. Line scanning was used to assess co-localization of the *green* fluorophore (Peg3) with the nuclear staining (*red*) as measured between the *white arrows*. Importantly, full-length Peg3 was the only condition where both channels superimposed, indicating co-localization of Peg3 and DNA (Fig. 1, *F–I*). These results were corroborated by biochemical cell fractionations, confirming that only full-length Peg3 was present in the nucleus (Fig. 1, *J–M*). These findings indicate that Peg3 is capable of entering the nucleus of PAER2 cells and co-localizing with DNA to potentially regulate transcription.

##### Endogenous Peg3 Localizes to the Nucleus following Autophagic Induction with Either Decorin or Rapamycin

**FIGURE 2.**
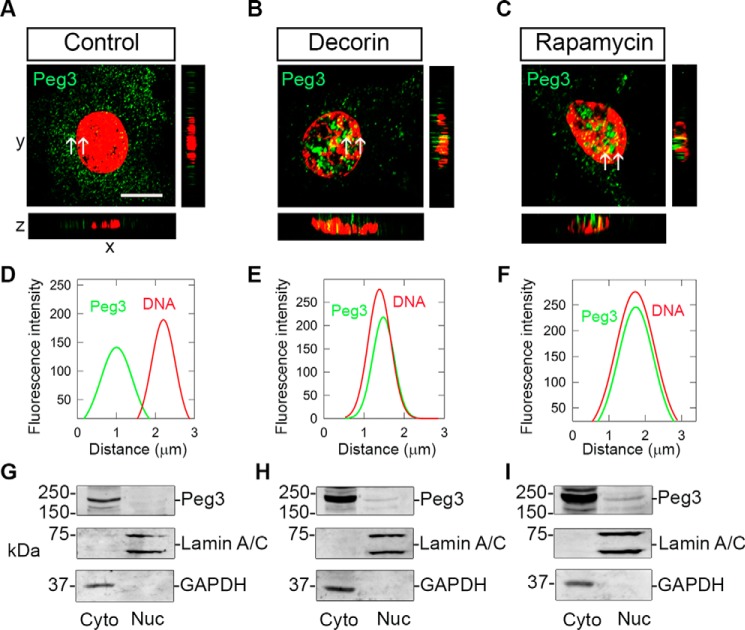
**Peg3 translocates to the nucleus following decorin- or rapamycin-induced autophagy.** *A–C*, confocal microscopy of endogenous Peg3 (*green*) present in the cytoplasm under basal conditions (*A*) and localized to the nucleus upon treatment with decorin (200 nm) (*B*) or rapamycin (40 nm) (*C*). Nuclei were stained with DAPI (false colored *red*) (*scale bar*, ∼10 μm). *D–F*, line scanning between the *white arrows* indicating co-localization of Peg3 with DNA. All images were captured using the same exposure, gain, and intensity. *G–I* cytoplasmic (*Cyto*) and nuclear (*Nuc*) fractionations performed to validate Peg3 cellular localization after decorin and rapamycin treatment as compared with basal conditions.

To determine whether endogenous Peg3 localizes to the nucleus in response to autophagic induction, PAER2 cells were treated with decorin or rapamycin, an established mTOR inhibitor. Under basal conditions, endogenous Peg3 resided primarily in the cytoplasm (Fig. 2*A*), but it efficiently translocated into the nuclei following decorin or rapamycin treatment (Fig. 2, *B* and *C*). Furthermore, line scanning of the areas between the *white arrows* demonstrates co-localization of Peg3 with DNA (Fig. 2, *D–F*) only after autophagic stimulation. Biochemical data using cytoplasmic-nuclear fractionation further confirmed that Peg3 was virtually undetectable in the nucleus under basal conditions but translocated after treatment of decorin or rapamycin (Fig. 2, *G–I*). These data validate the presence of endogenous Peg3 in the nucleus upon autophagic activation.

##### Peg3 Evokes BECN1 Promoter Activity

**FIGURE 3.**
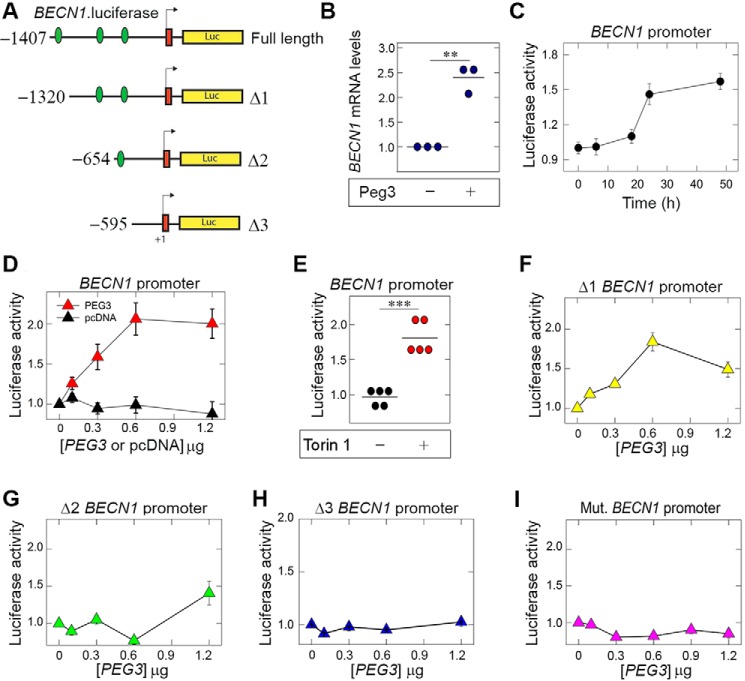
**Peg3 transcriptionally modulates *BECN1* activity.** *A*, full-length *BECN1* promoter containing three predicted binding sites (*green ovals*) and serial 5′ promoter truncations were inserted into a pGL3 Basic luciferase vector. *B*, effects of Peg3 expression on *BECN1* mRNA levels, normalized to *ACTB* mRNA. Values represent three independent trials performed in triplicate. *C*, time course of Peg3 transfections showing optimal luciferase activity of the full-length *BECN1* promoter at 48 h. *D–H*, representative luciferase reporter assays of PAER2 cells stably transfected with the indicated luciferase constructs and then transiently transfected for 48 h with the designated concentrations of Peg3, normalized to total protein. Significant values represent three independent trials performed in triplicate (***, *p* < 0.001; **, *p* < 0.01; *, *p* < 0.05 as compared with 0 ng of Peg3; Student's *t* test). Treatment of PAER2*^BECN1-Luc^* with the mTOR inhibitor Torin 1 was used as a positive control. *I*, *BECN1* promoter was synthesized by GenScript, incorporating nucleotide base changes, 5′-TGGCT-3′ to 5′-TAACC-3′, of the putative Peg3-binding site. The promoter region was inserted into a luciferase reporter construct and stably transfected into PAER2 cells. Transient transfection of Peg3 displayed no significant change in reporter activity (Student's *t* test; *p* > 0.05). Values represent three independent trials performed in triplicate. *Error bars* represent S.E. *Mut.*, mutant.

To investigate whether Peg3 does indeed regulate *BECN1* promoter activity in PAER2 cells, we utilized a vector harboring a 1.4-kb promoter region (here referred to as full length) of the *BECN1* gene and a series of 5′ deletion constructs fused to the luciferase reporter gene (Fig. 3*A*). We identified three putative binding sites for Peg3 encompassing the core sequence (5′-TGGCT-3′) within the 1.4-kb region of the *BECN1* promoter (76, 77). We found that cells constitutively expressing Peg3 had a 2-fold increase in *BECN1* mRNA vis-à-vis normal counterparts, suggesting regulation of Beclin 1 by Peg3 at the level of transcription (*p* < 0.01; Fig. 3*B*). We then generated cell lines stably expressing luciferase driven by the *BECN1* promoter and then transiently transfected these cells with increasing concentrations of a Peg3-containing expression vector. Time course experiments revealed that transient transfection of Peg3 was optimal at 48 h for robust luciferase activity of the full-length *BECN1* promoter (Fig. 3*C*). Using the full-length promoter, luciferase induction was dose-dependent and saturable with an initial increase at ∼100 ng and saturation occurring at ∼600 ng (Fig. 3*D*, *red triangles*). In contrast, transient transfection with equimolar amounts of empty vector had no effect (Fig. 3*D*, *black triangles*). Additionally, Torin 1, an ATP-competitive inhibitor of mTOR (78), induced *BECN1* promoter activity to levels nearly comparable with those achieved by Peg3 overexpression, validating that our luciferase reporter system increases activity under proautophagic conditions (*p* < 0.001; Fig. 3*E*).

To identify the minimal region for induction of *BECN1* promoter activity by Peg3, we used the 5′ truncation mutants whereby each promoter truncation lacked one predicted Peg3-binding site. Peg3 was able to promote a significant induction at 600 ng in the Δ1 *BECN1*-luciferase stable cell line that contained two predicted Peg3-binding sites (*p* < 0.001; Fig. 3*F*). Interestingly, after the second Peg3-binding site was eliminated (Δ2), Peg3 had no effect on luciferase activity (Fig. 3*G*), similar to the elimination of all three Peg3-binding sites (Δ3; Fig. 3*H*).

Additionally, to determine whether Peg3 directly associates at these putative binding sites within the *BECN1* promoter, we generated a 1.4-kb *BECN1* promoter-luciferase construct incorporating nucleotide changes to the core Peg3 binding consensus site. The core sequence (5′-TGGCT-3′) of the Peg3-binding site was mutated to 5′-TAACC-3′ for all three potential binding sites. The point mutations were verified through Sanger sequencing (data not shown). Notably, the PAER2 cells stably expressing the promoter with mutant Peg3-binding sites did not respond to increasing concentrations of transfected Peg3 cDNA (*p* > 0.05; Fig. 3*I*). Collectively, these data indicate that Peg3 mediates *BECN1* transcription and that the minimal *BECN1* promoter region required for Peg3-dependent expression lies between −1407 and −645 bp.

##### De Novo Expression of Peg3 Increases Beclin 1 Protein Levels in Endothelial Cells

**FIGURE 4.**
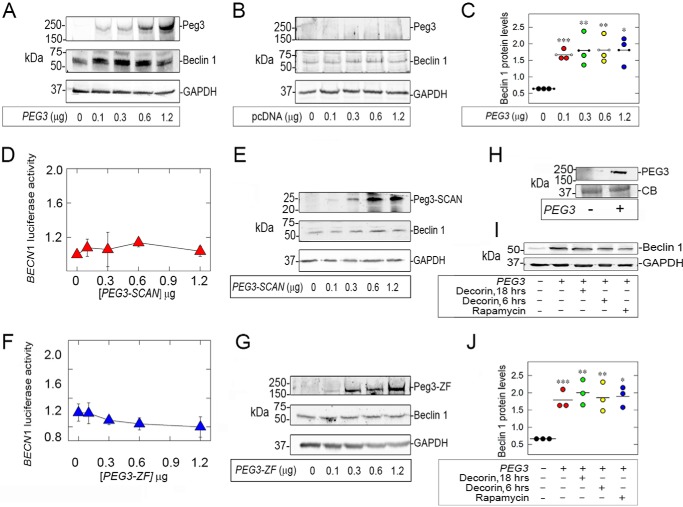
***De novo* overexpression of full-length Peg3 is necessary for increased *BECN1* transcription and protein expression in endothelial cells.** *A–C*, Western blots of endothelial cell lysates to validate transfection efficiency. Notice the dose-dependent increase in Peg3 and concomitant induction of Beclin 1 (*A*) as compared with transfection with empty vector (*B*). *C*, quantification of three independent experiments performed in triplicate (Student's *t* test; ***, *p* < 0.001; **, *p* < 0.0; *, *p* < 0.05). *D* and *F*, cells transfected with truncated Peg3 for 48 h display no significant change in *BECN1* promoter activity, normalized to total protein. *E* and *G*, representative Western blots validating transfection efficiency of truncations and confirming no effect on Beclin 1 protein expression. *H*, confirmation of stable overexpression of Peg3. *I*, representative Western blot of Beclin 1 in stably expressing Peg3 cells following treatment with decorin (200 nm) or rapamycin (40 nm) for the designated time points. *J*, quantification of three independent experiments (Student's *t* test; ***, *p* < 0.001; **, *p* < 0.01; *, *p* < 0.05). *Error bars* represent S.E.

Next, we assessed the effect on endothelial Beclin 1 protein levels evoked by increasing concentrations of Peg3 cDNA. The level of transgenic HA-tagged Peg3 was directly proportional to the transfected Peg3 cDNA (Fig. 4*A*) in contrast to empty vector (Fig. 4*B*). Notably, the levels of Peg3 protein correlated with induction of Beclin 1 protein (Fig. 4, *A* and *C).* Next, PAER2*^BECN1-Luc^* cells were transiently transfected with increasing concentrations of the truncated Peg3 constructs PEG3-SCAN and PEG3-ZF. Both Peg3 deletions failed to induce luciferase activity (Fig. 4, *D* and *F*), which was expected because neither truncation was capable of nuclear translocation (Fig. 1, *D* and *E*). Furthermore, these results correlated with protein levels of Beclin 1, which also remained unchanged (Fig. 4, *E* and *G*). Thus, the entire Peg3 protein is necessary for *BECN1* transcriptional induction and consequent protein expression.

To further evaluate Peg3-mediated Beclin 1 expression, we generated endothelial cells stably expressing full-length Peg3, PAER2*^PEG3^* (Fig. 4*H*), and found that basal levels of Beclin 1 were significantly up-regulated (Fig. 4*I*). We have previously shown that treatment of endothelial cells with decorin or rapamycin results in increased Beclin 1 protein expression (5). Interestingly, neither treatment further increased Beclin 1 levels in PAER2*^PEG3^* (Fig. 4, *I* and *J*). These findings suggest that Beclin 1 expression levels in Peg3-overexpressing cells were already maximal and could not be further enhanced by either decorin or rapamycin.

##### Peg3 Overexpression Promotes Autophagic Flux

**FIGURE 5.**
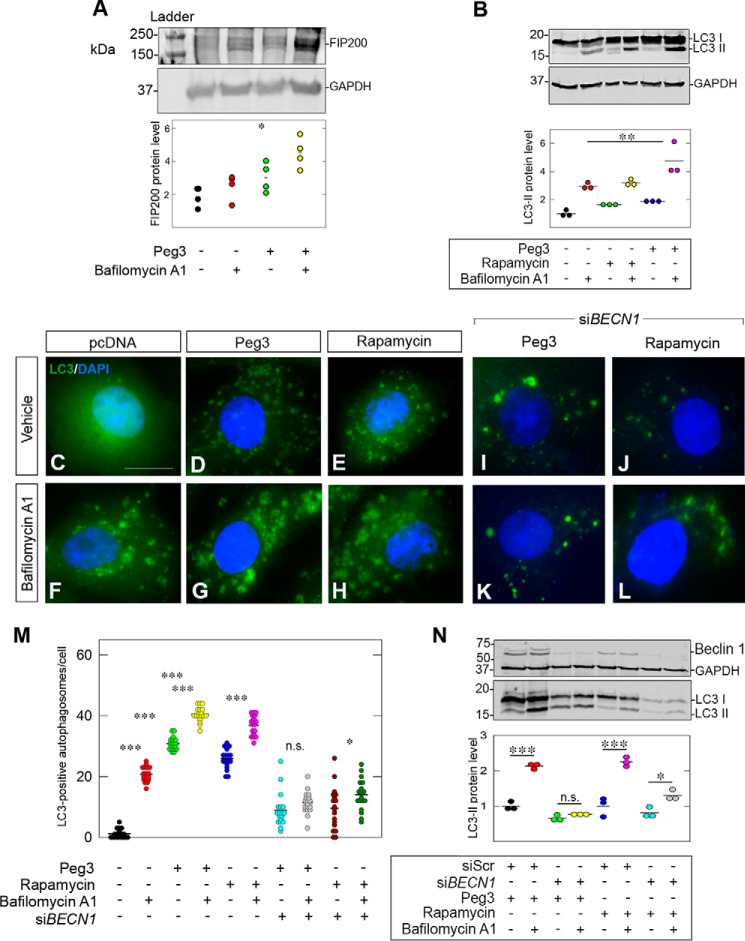
**Peg3 evokes Beclin 1-dependent autophagic flux.** *A*, representative Western blot of FIP200 following 6-h treatment with bafilomycin A1 (100 nm) of PAER2 cells stably transfected with either empty vector or Peg3. The Western blot is representative of four independent trials with similar results. Quantification of FIP200 shows statistical significance over empty vector control in the presence of bafilomycin A1 (Student's *t* test; *, *p* < 0.05). *B*, representative Western blot of LC3-II in PAER2*^pcDNA^*, PAER2*^PEG3^*, and PAER2*^pcDNA^* treated with rapamycin (40 nm) in the absence and presence of bafilomycin A1. Quantification of three independent experiments shows significant increases in LC3-II protein with bafilomycin A1 treatment in the presence of Peg3 compared with empty vector (Student's *t* test; **, *p* < 0.01). *C–H*, immunofluorescence images of PAER2*^GFP-LC3^* cells transfected with the designated vectors showing LC3 (*green*) puncta in the absence and presence of bafilomycin A1. Rapamycin was used as a positive control. *I–L*, immunofluorescence images of Beclin 1-deficient PAER2*^GFP-LC3^* cells following Peg3 overexpression and rapamycin treatment (*scale bar*, ∼10 μm). Images in *C–H* and *I–L* were obtained from separate experiments where each respective set of experiments utilized the same exposure, gain, and intensity. *M*, quantification of *C–L* (Student's *t* test; ***, *p* < 0.001; *, *p* < 0.05). *N*, biochemical analysis of LC3-II following Beclin 1 knockdown in cells overexpressing Peg3 or treated with rapamycin. Quantification is shown following three independent experiments (Student's *t* test; ***, *p* < 0.001; *, *p* < 0.05; *n.s.*, not significant).

To determine whether *de novo* Peg3 expression enhances autophagic flux following the transcriptional induction of Beclin 1, we treated the PAER2*^PEG3^* cells with bafilomycin A1. Bafilomycin A1 blocks the vacuolar (V-type) H^+^-ATPase, thereby inhibiting autophagosomal fusion with lysosomes, leading to a buildup of autophagic intermediates (16). This inhibition of autophagic flux allows for a better assessment of autophagic activity than any static time point as any proteins degraded by this process (*i.e.* LC3) will accumulate, permitting a more accurate quantitation of their turnover. Furthermore, we must note that LC3 and Beclin 1 are intermediates in converging lysosomal degradation pathways: LC3-associated phagocytosis, a process typically reserved for macrophages and a few other select cell types such as retinal pigment epithelial cells (79), and canonical autophagy. To determine which pathway was affected by Peg3, we tested the expression of FIP200, a specific autophagic marker (80). Indeed, we observed significant increases in FIP200 following Peg3 overexpression vis-à-vis empty vector in the presence of bafilomycin A1 (Fig. 5*A*), suggesting that Peg3 evokes turnover of FIP200. Thus, Peg3 specifically promotes canonical autophagy rather than LC3-associated phagocytosis.

During autophagy, LC3 is cleaved and conjugated with phosphatidylethanolamine to form LC3-II. This lipidated LC3-II associates with the outer membrane of the autophagosome and, as mentioned above, is itself degraded by the autophagic process. As such, it has been used as a reliable marker of autophagic flux (81, 82). PAER2*^PEG3^* cells displayed a higher level of LC3-II as compared with cells transfected with empty vector (Fig. 5*B*, compare *lanes 1* and *5*). These cells also demonstrated more LC3-II in the presence of bafilomycin A1 (Fig. 5*B*, compare *lanes 5* and *6*), suggesting that the increase in LC3-II compared with PAER2*^pcDNA^* in the absence of autophagic blockade was due to increased autophagic activity evoked by Peg3 overexpression. Importantly, treatment with bafilomycin A1 in conjunction with constitutive expression of Peg3 evoked a significant increase in LC3-II above that induced by bafilomycin A1 treatment alone (*p* < 0.01; Fig. 5*B*, compare *lanes 2* and *6*). Therefore, Peg3 increases LC3 turnover (and hence autophagic flux) beyond basal levels. This increase in LC3-II expression was similar to that seen with treatment of rapamycin in combination with bafilomycin A1.

Next, we used immunofluorescence to visualize autophagic flux. These findings mirrored the results seen at the biochemical level where PAER2*^GFP-LC3^* cells transfected with Peg3 showed an increase in LC3 puncta as compared with vehicle, similar to treatment with rapamycin (Fig. 5, *C–H*). Furthermore, more LC3-positive puncta were observed in cells overexpressing Peg3 compared with empty vector in the presence of bafilomycin A1, validating the hypothesis that Peg3 overexpression induces autophagic flux beyond levels seen in cells expressing endogenous Peg3.

To determine that Peg3 indeed utilizes the Beclin 1 pathway in the induction of autophagy, we silenced Beclin 1 using siRNA. Depletion of Beclin 1 in PAER2*^PEG3^* cells abolished Peg3-driven autophagic flux (Fig. 5, *I*, *K*, and *N*) where Beclin 1-deficient PAER2*^PEG3^* treated with bafilomycin A1 displayed no significant increase in LC3-positive puncta (Fig. 5, *I* and *K*) or LC3-II protein levels (Fig. 5, *M* and *N*) vis-à-vis PAER2*^PEG3^*. Moreover, as a positive control, PAER2*^pcDNA^* cells treated with rapamycin also displayed a decrease in autophagic flux when Beclin 1 was silenced (Fig. 5, *J*, *L*, *M*, and *N*). These data underscore the importance of the Peg3-Beclin 1 axis for competent autophagic flux and position Peg3 as a critical regulator of endothelial cell autophagy.

##### Peg3 Inhibits Endothelial Cell Motility and Emigration from a 3D Matrix

**FIGURE 6.**
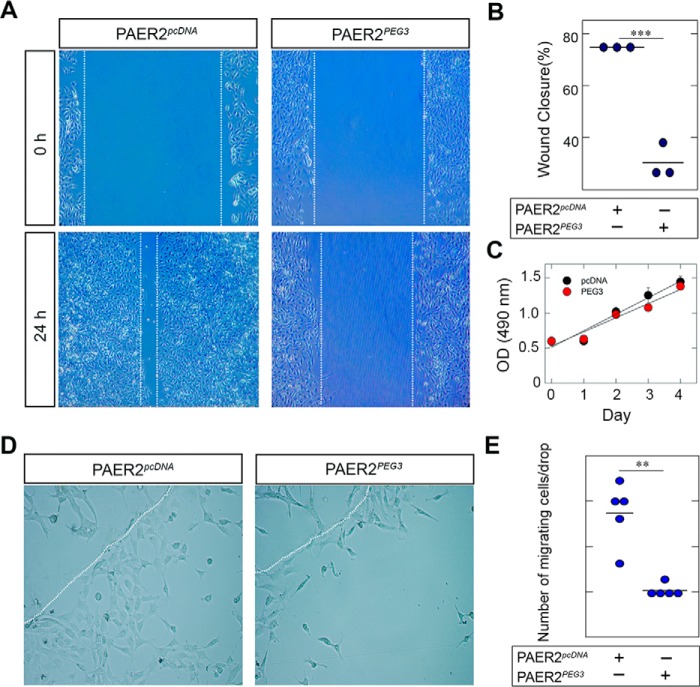
***De novo* Peg3 expression attenuates wound healing and evasion from 2D and 3D matrices.** *A*, motility assays of PAER2 cells overexpressing Peg3 or empty vector. Monolayers were uniformly scratched to form a wound, and images were taken at 0 and 24 h. *B*, quantification of percent wound closure at 24 h as compared with zero time (Student's *t* test; *n* = 3 each; ***, *p* < 0.001). *C*, MTT proliferation assays performed between empty vector- and PEG3-expressing cells over a period of 4 days. Results are expressed as absorbance at 490 nm and represent triplicate measurements from at least three independent experiments. *D*, evasion of PAER2*^pcDNA^* and PAER2*^PEG3^* cells embedded in a Matrigel matrix. *Dotted white lines* outline the edge of the Matrigel drops. Images were captured at 24 h. *E*, quantification of cells migrated from each Matrigel drop. Data are of five independent experiments (Student's *t* test; **, *p* < 0.01). *Error bars* represent S.E.

Both *in vivo* and *ex vivo* assays utilizing *Becn1*^+/−^ mice demonstrated increased angiogenic activity relative to wild-type mice (83). Notably, endothelial cells derived from *Becn1*^+/−^ mice display increased migration and tube formation, suggesting a link between Beclin 1 and regulation of angiogenesis. Therefore, we sought to determine whether overexpression of Peg3 would affect angiogenesis. In *in vitro* wound healing assays, endothelial cells stably expressing Peg3 were not able to close the wound as efficiently as compared with control cells expressing an empty vector (Fig. 6*A*). Indeed, PAER2*^pcDNA^* displayed an approximately ∼80% wound closure after 24 h, whereas PAER2*^PEG3^* displayed only a ∼25% wound closure after 24 h (*p* < 0.001; Fig. 6*B*).

To determine whether the inability of PAER2*^PEG3^* cells to close the wound was due to a decrease in motility or a decrease in proliferation, we performed MTT proliferation assays. We found no significant change in proliferation between cell types over a period of 4 days (Fig. 6*C*), indicating that Peg3 affects primarily endothelial cell motility.

To expand and corroborate the results obtained in a 2D system, we performed emigration assays where endothelial cells stably expressing Peg3 or empty vector were embedded in a 3D matrix composed of growth factor-reduced Matrigel. Following 24 h, PAER2*^PEG3^* cells had a markedly reduced number of cells that emigrated from the 3D matrix (Fig. 6*D*), and this was statistically significant (*p* < 0.01; Fig. 6*E*). Collectively, these data indicate that Peg3 inhibits endothelial cell motility in both 2D and 3D environments.

##### Peg3 Alters the Secretome to Inhibit Endothelial Cell Motility

**FIGURE 7.**
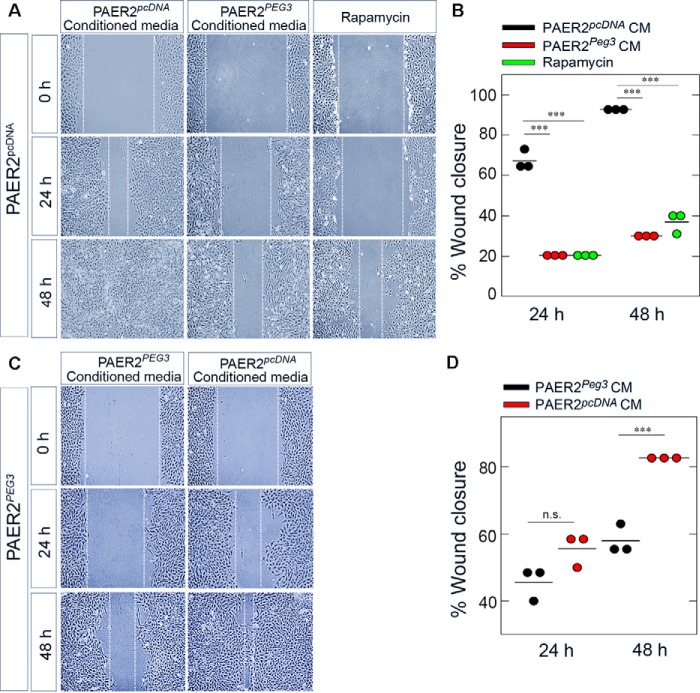
**Peg3 alters the secretome to inhibit wound healing.** *A*, motility assays of transgenic PAER2*^pcDNA^* cells incubated with PAER*^PEG3^*-conditioned media or treated with 40 nm rapamycin. *B*, quantification of percent wound closure after 24- and 48-h incubation with conditioned media (*CM*) or rapamycin as compared with time 0. Data represent three independent experiments (Student's *t* test; ***, *p* < 0.001). *C*, motility assay rescue experiments of PAER*^PEG3^* cells incubated with PAER2*^pcDNA^*-conditioned media. *D*, quantification of PAER2*^PEG3^* cells incubated with conditioned media. Data represent three independent trials performed in triplicate (Student's *t* test; ***, *p* < 0.001; *n.s.*, not significant).

To examine whether the decrease in wound closure was due to secreted factors evoked by Peg3 overexpression, we performed scratch assays using media conditioned for 48 h by PAER2*^pcDNA^* or PAER2*^PEG3^* cells. PAER2*^pcDNA^* cells incubated in their own conditioned media displayed an almost complete closure after 48 h, whereas PAER2*^pcDNA^* cells incubated in media conditioned by PAER2*^PEG3^* cells exhibited a significant reduction in wound closure at both 24 and 48 h (Fig. 7*A*). To provide a potential link to Peg3-mediated autophagy, treatment with rapamycin was utilized, which also inhibited wound closure to a similar extent as seen with the PAER2*^PEG3^*-conditioned media (*p* < 0.001; Fig. 7*B*).

To further validate that secreted factors contributed to the decrease in wound closure in the PAER2*^PEG3^* cells, we treated these cells with media conditioned by the PAER2*^pcDNA^* cells. Indeed, we were able to partially rescue the inhibitory effect of media conditioned by PAER2*^PEG3^* cells (Fig. 7*C*). After 48 h, there was a significant increase in wound closure when compared with PAER2*^PEG3^* cells incubated in their own conditioned media (*p* < 0.001; Fig. 7*D*). We conclude that Peg3 alters the endothelial cell secretome and may inhibit angiogenesis by modulating the secretion of antiangiogenic factors.

##### Peg3 Induces Thrombospondin 1 Secretion and Inhibits Capillary Morphogenesis Independently of Beclin 1

**FIGURE 8.**
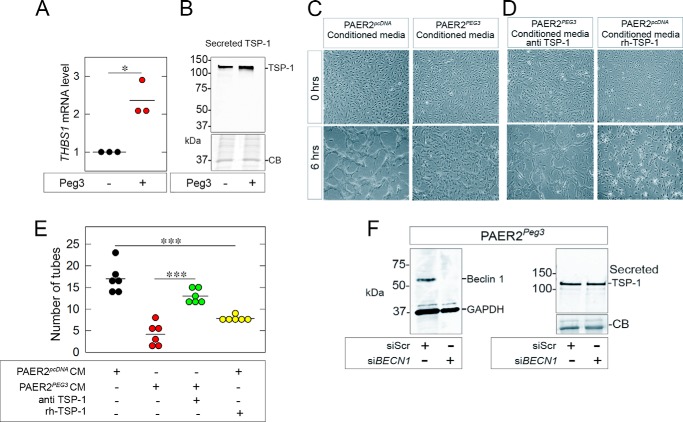
**Peg3 inhibits endothelial capillary morphogenesis via TSP-1 independently of Beclin 1.** *A*, PAER2*^PEG3^* cells have increased *THBS1* mRNA levels, normalized to *ACTB* mRNA. Values represent three independent experiments performed in triplicate (Student's *t* test; *, *p* < 0.05). *B*, Western blot of 48-h conditioned media from stable cell lines displaying an increase of TSP-1 secretion with constitutive expression of Peg3, normalized to cell number. *C*, HUVECs incubated with conditioned media and coated with 1 mg/ml fibrillar collagen I gel to investigate capillary morphogenesis. *D*, capillary morphogenesis was rescued by the addition of an anti-TSP-1-blocking antibody to the PAER2*^PEG3^*-conditioned media and inhibited by treatment with rh-TSP-1 (1 μg) added to the PAER2*^pcDNA^*-conditioned media. *E*, quantification of the number of tubes formed per captured image of capillary morphogenesis assays. Data are of three independent trials (Student's *t* test; ***, *p* < 0.001). *F*, immunoblot displaying efficient knockdown of Beclin 1 in PAER2*^PEG^*^3^ cells using 100 pm si*BECN1*; scrambled siRNA (*siScr*) was used as a control. Knockdown of Beclin 1 had no effect on TSP-1 secretion by PAER2*^PEG^*^3^. *CB*, Coomassie Blue; *CM*, conditioned media.

As decorin induces rapid Thrombospondin 1 (TSP-1) secretion in triple negative breast carcinoma cells (84), we hypothesized that the decorin-inducible Peg3 could be directly involved in stimulating the release and potential synthesis of TSP-1. This hypothesis was further strengthened by our close analysis of the *THBS1* promoter where we identified two putative Peg3-binding sites within a 3-kb promoter region. Notably, PAER2*^PEG3^* cells had a significant increase (*p* < 0.05) in *THBS1* mRNA expression as compared with PAER2*^pcDNA^* (Fig. 8*A*). Moreover, immunoblotting of media conditioned by Peg3-overexpressing endothelial cells showed enhanced TSP-1 secretion when normalized to cell number (Fig. 8*B*).

Next, we performed capillary morphogenesis assays in fibrillar collagen I using human umbilical vein endothelial cells (HUVECs). HUVECs incubated in media conditioned by PAER2*^pcDNA^* cells formed capillary-like structures after 6 h; in contrast, HUVECs incubated in media conditioned by PAER2*^PEG3^* failed to form tubes, further indicating that inhibition of capillary morphogenesis was due to an abundance of a secreted antiangiogenic factor (*e.g.* TSP-1) (Fig. 8*C*). Importantly, supplementing media conditioned by PAER2*^PEG3^* with a TSP-1-blocking antibody partially rescued tube formation. Furthermore, media conditioned by PAER2*^pcDNA^* treated with rh-TSP-1 abolished tube formation in HUVECs, mimicking the effects seen with the PAER2*^PEG3^*-conditioned media (Fig. 8*D*). Quantitatively, there was a 4-fold suppression in the number of tubes formed when cells were incubated with PAER2*^PEG3^*-conditioned media but significant tube formation when TSP-1 was blocked (*p* < 0.001; Fig. 8*E*).

Next, we determined whether the Peg3 induction of Beclin 1 was directly linked to the increase in TSP-1 secretion. Beclin 1 was knocked down in PAER2*^PEG3^* cells using siRNA, and the media were collected after 48 h (Fig. 8*F*). To our great surprise, we found that the level of TSP-1 secretion was not altered, indicating that Beclin 1 had no effect on the already augmented secretion of basal TSP-1 as evoked by the *de novo* and stable expression of Peg3 (Fig. 8*F*).

These findings provide robust evidence that the inhibition of endothelial cell motility evoked by Peg3 is due to an alteration of the endothelial secretome independent of Beclin 1. Thus, TSP-1 is a potent contributor to the antiangiogenic effect of Peg3.

## Discussion

We provide the first evidence that an imprinted gene is capable of inducing autophagy, a highly conserved eukaryotic process that maintains cellular homeostasis (16). Previous studies have implicated an aberrant autophagic pathway in several diseases including cancer and neurodegenerative and myodegenerative diseases (85–89). Basal autophagy is particularly important in tissues where cells are non-proliferative such as neurons and myocytes. In such tissues, fine-tuned cytosolic turnover is necessary for survival, and interestingly, these are regions in which Peg3 is highly expressed (86, 90). Moreover, Peg3 is induced upon starvation, a condition that activates autophagy (91).

In cancer, autophagy plays a dual role: it can function as a tumor suppressor, inhibiting tumor initiation through clearance of misfolded proteins, reactive oxygen species, and other factors that contribute to genomic instability. However, it can promote tumor cell survival by enabling cancer cells to overcome high energy demands (92–94). Notably, reintroduction of Peg3 into glioma xenografts inhibits tumor growth, suggesting that Peg3 functions as a tumor suppressor. Other established tumor suppressor genes (*i.e.* p53, phosphatase and tensin homolog (PTEN), death-associated protein kinase, and tuberous sclerosis 1 and 2), which are also silenced in many cancers, are capable of stimulating autophagy (92). In fact, expression of the proautophagic protein Beclin 1 correlates with cancer prognosis where low levels are associated with a worse outcome in colorectal, pancreatic, gastric, and breast cancers and high levels of expression are associated with improved survival (95–97). Our data provide evidence that Peg3 modulates *BECN1* expression to evoke autophagy in endothelial cells. This may contribute to tumor growth inhibition by suppressing angiogenesis as well as by promoting autophagic cell death considering that Peg3 also functions downstream of p53 to induce apoptosis and these two pathways are interconnected (98). Furthermore, HUVECs treated with the angiogenesis inhibitor bortezomib undergo autophagic cell death (99).

Previous studies have shown that Peg3 is primarily localized to the nucleus where it regulates a subset of genes involved in development and differentiation (24, 30, 100). Breeding experiments using a Peg3 mutant mouse model have proven that Peg3 transcriptionally regulates placenta-specific genes in the brain and genes involved in lipid metabolism (101, 102). Under basal, unstimulated conditions, Peg3 is primarily located in the cytoplasm of endothelial cells (5). We expected that both the full length and the zinc finger-containing domain would translocate to the nucleus as both harbor a nuclear localization signal. Surprisingly, only the full-length Peg3 was capable of nuclear translocation. It is possible that the SCAN domain is necessary for specific protein-protein interactions that allow Peg3 to enter the nucleus.

In this study, we find that *BECN1* is a novel Peg3 target gene and identify a minimal promoter region between −1407 and −654 containing two Peg3-binding sites. The increase in *BECN1* transcription upon Peg3 overexpression is concomitant with the induction of protein levels of Beclin 1. Additionally, we present evidence that Peg3 directly modulates *BECN1* activity as mutation of putative Peg3-binding sites within the *BECN1* promoter region abolishes luciferase activity. Notably, in mouse brain, Peg3 can directly bind the promoter region of phosphoglucomutase 2-like 1 (*Pgm2l1*), the mouse homolog of glucose-1,6-biphosphate synthase, via the Peg3 binding motif. Thus, Peg3 is capable of directly binding DNA (76).

Endothelial cells stably expressing Peg3 display an increase in LC3-II, the lipidated form of LC3. This confirms our previous results that Peg3 functions within the PI3K/Akt/mTOR pathway (5). Importantly, treatment with bafilomycin A1 demonstrates that Peg3 induces autophagic flux. If autophagy induction by Peg3 were due to an inhibition of protein degradation, bafilomycin A1 treatment would have had no effect on LC3-II levels. Indeed, silencing Beclin 1 abrogates autophagic flux in Peg3 stably transfected cells, corroborating that Beclin 1 is necessary for Peg3-induced autophagy. Furthermore, although FIP200 has not yet been reported to be a substrate of autophagy, we show for the first time that Peg3-induced autophagy clears FIP200 in endothelial cells. This finding suggests new avenues of exploration for the nuances of Peg3-mediated autophagic control.

Inhibition of autophagy by knockdown of the autophagic gene *ATG*7 has been shown to stimulate cell migration (103). It has also been demonstrated that decorin, an inducer of autophagy, is capable of blunting capillary morphogenesis and cell migration (51, 64) and interacting with various metalloproteinases (70) involved in modulating angiogenesis, wound repair, and fibrosis (34, 104–107). Recently, this ability of decorin to inhibit migration has been directly linked to its induction of autophagy (108). Peg3 functions downstream of decorin in the induction of autophagy in endothelial cells, and here we provide further evidence that Peg3 also blunts cell migration in both 2D and 3D environments. We must emphasize that Peg3 is positioned in an extracellularly regulated signaling axis where it is a direct downstream target of decorin and endorepellin, two soluble matrix constituents that both halt angiogenesis by interfering with VEGFR2 (5, 9, 10, 109–112). Thus, there is a likely possibility of a connection among Peg3, autophagy, and angiogenesis.

Our study also provides mechanistic evidence that Peg3 inhibits motility and capillary morphogenesis by promoting the secretion of TSP-1, a powerful antiangiogenic factor (113–115). Although our aim was to connect TSP-1 secretion to Peg3-induced autophagy, we found that this secretion occurred independently of Beclin 1. We must reiterate, however, that there are Peg3-binding sites in the proximal region of the *THBS1* promoter suggesting that, like *BECN1*, *THBS1* may be a direct Peg3 target gene. Interestingly, activation of the TSP-1 receptor, CD47, induces autophagy in RAS-expressing cancer cells to quell tumor growth (116). Thus, it is possible that the Peg3-induced secretion of TSP-1 may be an indirect pathway through which Peg3 mediates Beclin 1 expression (potentially via CD47) and subsequently autophagy. This process could potentially explain why loss of Beclin 1 does not affect TSP-1 secretion. Paradoxically, other studies illustrate that blocking CD47 inhibits autophagy (117, 118), suggesting that Peg3-mediated TSP-1 secretion may also act as a feedback mechanism to maintain homeostasis under the highly autophagic conditions promoted by Peg3 overexpression. Regardless of the situation, the relationship between Peg3 and TSP-1 has an important implication in autophagic control in endothelial cells and is something to be investigated in future studies.

Although TSP-1 secretion is a partial mechanism for Peg3-mediated angiostasis, other secreted bioactive antiangiogenic and proautophagic factors may be at play as well. We hypothesize that top candidates may be endostatin and endorepellin, both of which are synthesized and secreted by endothelial cells (74, 84). In particular, both endostatin and endorepellin are known upstream effectors of Beclin 1 (9, 119), thereby providing a potential connection among Peg3, angiogenesis, and autophagy.

In conclusion, the ability for Peg3 to evoke a vital intracellular catabolic process in endothelial cells along with its alteration of the endothelial secretome, resulting in restricted migration and blunted capillary morphogenesis, underscores the importance of this decorin-induced gene in the regulation of endothelial cell homeostasis. Future work will likely elucidate the intricacies of Peg3 in angiostasis in terms of autophagic regulation. These findings are merely the beginning and should provide new avenues for better understanding angiogenesis in the context of cancer.

## Experimental Procedures

### 

#### 

##### Antibodies, Cells, and Reagents

The rabbit polyclonal antibodies against human lamin A/C, GAPDH, and Beclin 1 were from Cell Signaling Technology (Danvers, MA). Rabbit monoclonal antibody against the HA tag was also from Cell Signaling Technology. Peg3 antibody was custom made at GenScript. HRP-conjugated goat anti-rabbit secondary was from Millipore, Inc. (Billerica, MA) Donkey anti-rabbit secondary (Alexa Fluor 488) was from Life Technologies. SuperSignal West Pico chemiluminescence substrate was from Thermo Fisher Scientific (Philadelphia, PA). HUVECs were grown in basal medium supplemented with VascuLife EnGS LifeFactors kit (LifeLine Cell Technology, Frederick, MD) with cells being utilized within the first five passages. Transgenic porcine aortic endothelial cells expressing VEGFR2 were described previously (120). These cells were stably transfected with a luciferase reporter construct driven by a 1.4-kb region or fragments containing a 1.3-kb, 645-bp, or 595-bp region of the *BECN1* promoter linked to 514 bp of the first exon and a portion of the first intron of the *BECN1* gene. Cells were grown at 37 °C in a 5% CO_2_ atmosphere in Dulbecco's modified Eagle's medium (DMEM) containing 4.5 g/liter glucose, l-glutamine, and sodium pyruvate from Life Technologies and supplemented with 10% fetal bovine serum (FBS) from Thermo Fisher Scientific and 100 units/ml penicillin/streptomycin from Life Technologies. Lipofectamine LTX and hygromycin B were from Invitrogen. Rapamycin was from Sigma-Aldrich.

##### Immunofluorescence and Confocal Microscopy

PAER2 cells (∼5 × 10^4^) were grown on coverslips coated with 0.2% gelatin. Cells were transfected with HA-PEG3, HA-SCAN, or HA-ZF for 48 h, then fixed with 4% paraformaldehyde at 4 °C, and permeabilized with 0.01% Triton X-100. Cell were blocked in 5% BSA in PBS, incubated with primary rabbit anti-HA antibody for 1 h at room temperature, and then incubated with donkey anti-rabbit Alexa Fluor 488 secondary antibody for 1 h. DAPI (Vector Laboratories) was used to visualize nuclei. Immunofluorescence and confocal (121–123) images were obtained as described previously (9).

##### Nuclear and Cytoplasmic Fractionation and Immunoblotting

Approximately 10^7^ transfected cells were harvested and centrifuged at 500 × *g* for 5 min. Cell pellets were washed in PBS, and fractionation was performed using NE-PER Nuclear and Cytoplasmic Extraction Reagents (Thermo Scientific). Nuclear pellets were washed twice with PBS to eliminate cytoplasmic contaminants before extraction. Following treatments, endothelial cells were lysed in radioimmune precipitation assay buffer (50 mm Tris-HCl, 50 mm NaCl, 1 mm EGTA, 1 mm EDTA, 1% Triton X-100, 0.5% sodium deoxycholate, 0.5% SDS, 1 mm sodium orthovanadate, 1 μg/ml leupeptin, 1 μg/ml aprotinin, 100 μm tosylphenylalanyl chloromethyl ketone, 1 mm PMSF, and one EDTA-free protease inhibitor tablet) for 20 min on ice. Proteins were separated by SDS-PAGE, transferred to nitrocellulose membrane (Bio-Rad), incubated with the appropriate antibodies, and visualized using enhanced chemiluminescence (Thermo Scientific) and an ImageQuant LAS 4000 (GE Healthcare).

##### Luciferase and Proliferation Assays

PAER2 cells were stably transfected with the indicated *BECN1*-luciferase constructs (primer sequences are listed in Table 1) and selected for 3 weeks with 500 μg of hygromycin B (Invitrogen). Mass cultures were collected and transiently transfected with increasing concentrations of Peg3 in 24-well plates. Luciferase was detected using a *Renilla* Luciferase Assay kit (Biotium) and measured using a plate luminometer (PerkinElmer Life Sciences). Data were normalized to total cell protein.

**TABLE 1:** **Primer sequences used for the construction of the indicated vectors** “F” denotes forward, and “R” denotes reverse.

Name	Primer sequence (5′ to 3′)
PEG3-SCAN	F, GCTAGCATGTACCATACGATGTTCCAGATTACGCTCTTCTGCCTCCAAAGCACTTG
	R, CTCGAGTGGTTGTACATCTCCTTGTAATTCCTCCAGCAGAGT
PEG3-ZF	F, GCTAGCATGTACCATACGATGTTCCAGATTACGCTCTTACGCAGGGCCACTCA
	R, GGATCCTCAGCCAGTGTGGGTATTCTGGTGTCTGGCGAGGGA
BECN1	F, GCTAGCTTTTGGGTTAAGCAGTGGTTTCTT
	R, CTCGAGTGAGGCCGTGGAAAAGAGGCAA
Δ1 BECN1	F, GCTAGCTTGGCTCACACCTGTAATCTCA
	R, CTCGAGTGAGGCCGTGGAAAAGAGGCAA
Δ3 BECN1	F, GCTAGCTGGTCTCGAACTCCTGACCTT
	R, CTCGAGTGAGGCCGTGGAAAAGAGGCAA

For cell proliferation assays, CellTiter Aqueous One Solution Cell Proliferation Assay was used (Promega). PAER2*^pcDNA^* and PAER2*^PEG3^* cells were seeded on 96-well microplates at a density of 5,000 cells/well in 100 μl of media. One Solution Reagent was added to the wells to be measured and incubated at 37 °C for 3 h each day for 4 days. Absorbance at 490 nm was recorded using a 96-well plate reader (PerkinElmer Life Sciences).

##### In Vitro Wound Healing, Matrigel Evasion, and Tube Formation Assays

For wound healing assays, PAER2 cells stably transfected with pcDNA or Peg3 were cultured on a 0.2% gelatin-coated 12-well dish. When cells reached subconfluence, scratches were made using a P-200 pipette tip. To evaluate the effect of conditioned media on wound healing, selected wells were incubated with 80% PAER2*^pcDNA^* or PAER2*^PEG3^* 48-h conditioned media. Tube formation assays were performed using HUVECs seeded on collagen-coated (100 μg/ml) 12-well dishes. After 24 h, 1 mg/ml fibrillar collagen (seven parts 1.4 mg/ml collagen, one part 10× medium 199, and two parts 11.8 mg/ml sodium bicarbonate) was placed on top of HUVECs and allowed to polymerize at 37 °C for 20 min. Conditioned media were placed over collagen gel, and select wells were treated with anti-Thrombospondin 1 (Santa Cruz Biotechnology) or rh-Thrombospondin 1 (R&D Systems) for 6 h. Conditioned media were collected after 48 h from confluent 10-cm dishes (10 × 10^6^ cells) and filtered through a disposable 0.22-μm syringe-driven filter unit (Millipore). Images were taken using a digital microscope camera (Leica D-LUX3). For the evasion assay, the stable transfected endothelial cells used above were mixed in 1:2 ratios with Matrigel (BD Biosciences), and drops were placed at the corners of gelatin-coated chamber glass slides. Images were captured 24 h after incubation at 37 °C in basal medium (Lifeline Cell Technologies) with a digital epiluminescence microscope camera (CKX41, Olympus).

##### Real Time Gene Expression and Analysis

Stable cell lines PAER2*^pcDNA^* and PAER2*^PEG^*^3^ were subjected to quantitative real time polymerase chain reaction (PCR) to confirm differences in *BECN1* and *THBS1* gene expression when Peg3 is constitutively expressed. Cells were lysed in 1 ml of TRIzol, and RNA was isolated using a Direct-zol RNA Miniprep kit (Zymo Research). Total RNA (1 μg) was annealed with oligo(dT) primers, and cDNA was synthesized using SuperScript Reverse Transcriptase II (Life Technologies). Gene-specific primer sets for *Sus scrofa* mRNA were designed for use in quantitative real time PCR. Target genes and endogenous housekeeping gene *ACTB* amplicons were amplified and analyzed as described previously (1).

##### siRNA-mediated Knockdown

Transient knockdown of Beclin 1 in PAER2*^PEG^*^3^ was achieved via 48-h transfection of siRNA specific for *S. scrofa* Beclin 1. Scrambled siRNA was used as a negative control. Two 10-cm dishes were seeded with PAER2*^PEG^*^3^ cells to achieve 80% confluence after attachment and transfected after 24 h with 100 pm scrambled siRNA or si*BECN1* and 10 μl of Lipofectamine RNAiMAX (Life Technologies) diluted in Opti-MEM medium (Gibco). Conditioned media were collected 48 h after transfection, and cells were lysed. Verification of siRNA-mediated knockdown was confirmed via Western blotting. Aliquots of conditioned media were analyzed by Western blotting to determine secreted levels of TSP-1 and normalized to cellular protein.

## Author Contributions

R. V. I. and A. T. designed the study. R. V. I., A. T., and M. A. G. analyzed the data and wrote the manuscript. A. T. and M. A. G. performed the research.

## Footnotes

3 The abbreviations used are:

Peg3: paternally expressed gene 3
AMPK: adenosine monophosphate-activated protein kinase
HUVEC: human umbilical vein endothelial cell
LC3: microtubule-associated light chain 3
mTOR: mammalian target of rapamycin
VEGFR2: vascular endothelial growth factor receptor 2
PAER2 cells: porcine aortic endothelial cells stably expressing VEGFR2
ZF: zinc finger domains
TSP-1: Thrombospondin 1
rh: recombinant human
BECN1: Beclin 1
MTT: 3-(4,5-dimethylthiazol-2-yl)-5-(3-carboxymethoxyphenyl)-2-(4-sulfophenyl)-2*H*-tetrazolium.
